# Evaluation of postural balance in postmenopausal women and its relationship with bone mineral density- a cross sectional study

**DOI:** 10.1186/1471-2474-13-2

**Published:** 2012-01-16

**Authors:** Luciana Mendes Cangussu, Jorge Nahas-Neto, Eliana Aguiar Petri Nahas, Ana Beatriz Cesar Rodrigues Barral, Davi de Araujo Buttros, Gilberto Uemura

**Affiliations:** 1 Department of Gynecology and Obstetrics, Botucatu School of Medicine, Sao Paulo State University - UNESP, Botucatu, Sao Paulo, Brazil

**Keywords:** Menopause, Postural Balance, Bone Mineral Density, Falls

## Abstract

**Background:**

Low bone mineral density (BMD) and falls are common problems encountered in the postmenopausal women. The purpose was to evaluate the association between postural balance and BMD in postmenopausal women and its relation to risk for falls.

**Methods:**

In this cross-sectional study, 225 women in amenorrhea > 12 months and age ≥ 45 years were included and divided, according to BMD, in T-score values > -2.0 SD (n = 140) and ≤ -2 SD (n = 85). Those with neurological or musculoskeletal disorders, history of vestibulopathies, uncorrected visual deficit or drug use that could affect balance were excluded. History of falls (last 24 months), clinical and anthropometric characteristics were evaluated. Postural balance was assessed by stabilometry (force platform). For statistical analysis were used Wilcoxon's Test, Chi-Square Test and logistic regression method for fall risk (Odds Ratio-OR).

**Results:**

Patients with BMD > -2.0 SD were younger, with shorter time since menopause, and showed higher BMI as compared to those with low BMD (≤ -2 SD) (p < 0.05). It was observed that 57.8% of the participants reported fall episodes without significant difference distribution between the groups (p = 0.055). No differences were found from the comparison between the groups (p > 0.05) for stabilometric parameters. Risk for falls increased with age (OR 1.07; CI 95% 1.01-1.13), current smoking (OR 2.19; CI 95% 1.22-3.21) and corrected visual deficit (OR 9.06; CI 95% 1.14-4.09). In contrast, hormone therapy (HT) use was significantly associated with reduced risk for falls (OR 0.48; CI 95% 0.26-0.88).

**Conclusions:**

In postmenopausal women, BMD did not show association with postural balance or risk for falls. Age, smoking and corrected visual deficit were clinical indicators of risk for falls whereas HT use showed to be a protective factor.

## Background

Postural stability and balance decrease with age. Loss of balance and increased body sway are important risk factors for falls in the postmenopausal women [1]. The age-associated increase in the incidence of osteoporotic fractures results from a combination of increased fall risk and reduced bone strength. Although various factors are associated with falls, impaired balance and mobility have been consistently identified as the main risk factors [2]. In fact, approximately one-third of women older than 60 years fall at least once a year [3]. Fall prevention should be routine in the management for postmenopausal women. Relevant clinical factors include fall history, weakness, fainting, sarcopenia, dizziness or vestibulopathies, mobility disabilities, arthritis or arthrosis, sedatives, and visual deficit [4]. Reduction in fall risk is focused on exercises aiming to improve balance and muscle strength, adjusting medication use and diminishing the danger for falls at home [5].

Falls resulting in fractures to postmenopausal women may seriously hinder quality of life and lead to high morbidity and mortality, as well as an increase in direct costs for health services [6]. Fractures have multifactor causes, however low bone mineral density (BMD) is considered to be a determinant factor [7]. In early post menopause, rapid bone mass loss occurs in response to hypoestrogenism. BMD reduction and structural integrity deterioration result in increased risk for osteoporosis in women [8]. Although the muscular and the skeletal systems are structurally interdependent, in women with low BMD, muscle conditions change, thus altering posture, and the center of gravity dislocates and hinders balance [9,10], which could lead to increased risk for falls. However there are few studies evaluating the impact of low bone mineral density with the postural sway in postmenopausal women [11-13].

Given the context of falls and their outcomes in the lives of postmenopausal women, efforts have been made in order to evaluate balance. By means of instruments for postural balance assessment, it is possible to identify women who are prone to falls, a major cause of functional loss and dependence, providing information regarding on performance level and on intervention needs [14]. Hence, considering imbalance is one of the major causes of falls and their resulting osteoporotic fractures, this study aimed at evaluating the association between postural balance and bone mineral density (BMD) in postmenopausal women and at correlating it with risk for falls.

## Methods

### Study design

This is a clinical, analytical, cross-sectional study. The population group (n = 225) consisted of patients seeking healthcare at a public outpatient center in Southeastern Brazil of the Botucatu Medical School from March 2009 to July 2010. The sample was non-probabilistic and by convenience. The sample size was based on the mean number of annual appointments realized at the Climacterium Outpatient Service which met the criteria proposed by the study. Women with at least 12 months of amenorrhea and age ≥ 45 years were included in the protocol. The non-inclusion criteria were: (1) presence of neurological diseases with balance alteration; (2) musculoskeletal disease with deformity of lower extremities; (3) presence of cognitive impairment; (3) uncorrected visual deficit; (4) clinical symptoms of dizziness, tinnitus, hypoacusis and auricular plentitude (uncontrolled vestibulopathies); (5) consumptive syndrome; (6) uncontrolled hypertension; (7) postural hypotension; (8) ingestion of medication that can change balance (sedative and hypnotic agents); (9) alcoholism; (10) grade-III obesity. All subjects were from low socioeconomic groups (income ≤ US$ 500). Informed consent was obtained from all subjects, and the study was approved by the Research Ethics Committee of the Botucatu Medical School-UNESP.

### Clinical data evaluation

The following data were initially collected on the day of consultation by means of interviews: age, age of menopause, time of menopause, parity, current smoking status, use or not of hormone therapy (HT), visual acuity, history of vestibulopathies and of chronic diseases (hypertension, diabetes, cardiovascular diseases, osteoarticular diseases), use of medication (bisphosphonates), personal history of fractures, physical activity, blood pressure, weight and height. According to the participants' history, the occurrence and sites of falls in the past 12 months were observed. A fall was identified as an unexpected position change which is unintentional and makes an individual remain in a lower level in relation to the initial position; it is not the result of sudden paralysis, an epileptic crisis or an external force [4]. Blood pressure was measured on the right arm with the forearm in rest at the level of the precordium, the palm of the hand facing upwards, using a standard aneroid sphygmomanometer and the patient in the sitting position. Women who did 30 min of physical aerobic exercise of moderate intensity at least five times a week or strength exercises three times a week were considered to be active [15]. Visual acuity was evaluated by means of subjective analysis using questions about the interference of vision in their daily lives and the use or not of glasses. The musculoskeletal deformities were excluded from the study clearly commit to the orthostatic position of the patient. Thus, all patients underwent a postural evaluation by a trained physiotherapist through a general visual inspection, where musculoskeletal deformities were identified. The patients who presented significant deformities such as anatomic alterations in the feet, acute dorsiflexion of knees, exacerbated posterior inclination of the pelvis, were not included in the study.

The following data were collected for anthropometric evaluation: weight, height, body mass index, waist and hip circumference and waist-hip ratio (WHR). An electronic anthropometric microdigital platform-type scale (Filizola^®^, Brasil), with a capacity of 150 kg and precision of 0.1 Kg and 0.5 cm (weight and height, respectively) was used for weight measurement. Weight was determined with the individual standing on the scale barefoot and wearing minimum clothing. For height measurement, the patient also remained barefoot, holding his/her arms along his/her body in the upright position and keeping his/her eyes fixed on a horizontal plane parallely to the floor. Height was measured by a vertical 0.5-cm grading ruler which was coupled to the scale. The 2002 World Health Organization criteria were used to classify patients according to BMI: smaller than 18.5 kg/m2 as low weight, from 18.5 to 24.9 kg/m2 as normal, from 25 to 29.9 kg/m2 as overweight, from 30 to 34.9 kg/m2 as grade-I obesity I, from 35 to 39.9 kg/m2 as grade-II obesity and greater than or equal to 40 kg/m2 as grade-III obesity. For waist measurement, the smallest circumference between the last rib and the antero-superior iliac crest was considered, and reading was performed at the moment of exhalation. It was considered to be increased for women when it was over 88 cm [16].

### Bone mineral density (BMD) assessment

Bone mineral density was assessed in all the participants. BMD was measured by dual energy X-ray absorptiometry (DXA) on the lumbar spine (L1 to L4) and femoral neck using a Hologic QDR-2000 (Waltham, MA, USA) device. The technique is based on the attenuation, by the patient's body, of a generation beam generated by an X-ray source with two energy levels. The variation coefficients in the testing period were lower than 2%. The values were classified by the T-score corresponding to the mean BMD value of normal young women minus the patients' BMD, divided by the standard deviation (SD) of the mean for normal young women. The participants with total-spine and/or femoral-neck T-score values ≤ -2.0 SD were considered to have low BMD [17].

### Balance evaluation

After the initial interview, the patients using HT or not were invited to participate in balance evaluation by means of stabilometry (force platform). This exam was performed in a silent environment, in a room specially designed for clinical evaluation, thus preventing patients from being distracted during the assessment. Prior to performing the test, the woman remained in the sitting position and in rest for 5 minutes. The clinical interview data were collected by the medical researchers (EAPN, JNN), whereas the balance evaluation data were taken by the same evaluator, blinded to clinical date (LMC).

Stabilometry is a technique used for evaluating balance in the orthostatic posture. It consists in quantifying anteroposterior and lateral oscillations of the body while an individual stands on a force platform [18]. The FootWork model (IST Informatique) of a force platform was used with a computer that records the dislocations of the center of pressure (CP) on the center of the platform in the antero-posterior (Y) and lateral (X) directions by means of a force exerted on the platform by the bottom of the feet and captured by the FootWork software (version 2.9.9.0). Once a CP dislocation is representative of postural oscillations, it is recorded by the instantaneous estimation of its position (x and y coordinates), which corresponds to the location of the resultant of the forces applied on the surface in contact with the feet on the support [19]. The frequency of acquisition of the CP signal depends on the task being investigated. Higher frequencies were used for the still upright position, which were around 100 Hz due to the frequencies of the noise present in the signal. The signals on the platform are captured by three charge transducers on its surface and recorded by a microcomputer coupled to the platform. The stabilometric parameters analyzed were: mean amplitude of the CP dislocation on the lateral plane (X axis, cm); mean amplitude of the CP dislocation on the antero-posterior plane (Y axis, cm); elliptic area of the CP dislocation on the platform plane (cm^2^) and sway velocity (cm/s) [19]. During examination, the participant was requested to adopt the orthostatic position on the platform, with bipodalic support, bare and aligned feet and heels separated by 2 cm, thus forming a 30° angle without surpassing shoulder alignment, relaxed arms along the body, looking fixedly at a target ahead and keeping her eyes open for 2 min. As regards the number of attempts, three repetitions were performed as recommended [19], in which the mean of the results was used.

### Statistics

From the collected data, tables containing the variables that influenced risk for balance alteration according to bone mineral density (< -2.0 SD e ≤ -2.0 SD) were designed. For data analysis, the medians and the 25th and 75th percentiles were estimated for quantitative variables, and percentages were calculated for qualitative variables. Homogeneity between the groups in relation to socio-demographic and anthropometric characteristics was evaluated by the Wilcoxon's Test, and the Chi-Square Test was used for the frequency of categorical clinical characteristics. The Wilcoxon's Test was utilized for postural balance parameter. Multivariate analysis was performed by binary logistic regression with a level of significance of *p *< 0.05 and a confidence interval of (CI) of 95%, and the respective odds ratio (OR) was estimated in order to observe the possible associations between risk for falls (dependent variable) and the influential variables of risk (independent variables). All clinical (age, age and time of menopause, BMI, WC, vision, physical exercise, smoking status, HT, BMD, foot size) and postural variables were tested by adjusting the multiple logistic regression model using the stepwise procedure for the variables showing significant difference. Only the statistically significant results were presented. The statistical tests were bilateral, and the level of significance adopted was of 5%. Statistical analyses were performed by the Statistical Analyses System (SAS), version 9.2.

## Results

The clinical, anthropometric and postural characteristics of postmenopausal patients divided according to bone mineral density in > -2.0 SD (n = 140) and ≤ -2 SD (n = 85) were statistically compared and are represented in Tables 1, 2, 3. In Table 1, it is observed that the groups were homogenous for the following variables: age of menopause, parity, foot size and waist-hip ratio (WHR). Statistically significant differences were observed as regards age, time of menopause, BMI and waist circumference. Patients with BMD > -2.0 SD were younger and had been menopausal for a shorter period of time. They also showed higher BMI with abdominal fat deposition (> WC) as compared with those with BMD ≤ -2 SD (*p *< 0.05). We observed that 24.4% of participants reported using bisphosphonates to treat osteoporosis, with an average of 2 years of use.

**Table 1:** Comparison of clinical and anthropometric characteristics in postmenopausal women (n = 225), according to bone mineral density (BMD)

Variables	BMD > -2.0 SD (n = 140)	BMD ≤ -2.0 SD (n = 85)	*p*-value*
Age (years)	54.0 (50.0/57.5)	58.0 (52.0/64.0)	**< 0.001**
Age of Menopause (years)	48.0 (43.5/50.0)	48.0 (42.0/52.0)	0.704
Menopause Duration (years)	7.0 (3.0/125.0)	10.0 (5.0/17.0)	**< 0.001**
Parity (no. of children)	2.0 (2.0/3.0)	3.0 (1.0/4.0)	0.318
Foot Size	37 (36/37)	36 (35/37)	0.058
BMI (kg/m^2^)	28.5 (25.7/32.3)	26.7 (23.1/29.9)	**< 0.004**
Waist (cm)	89.0 (81.0/98.0)	86.0 (77.0/90.0)	**0.001**

*SD *standard deviation; *BMI *Body Mass Index

Values expressed as median with the 25th and 75th percentiles in parentheses

*Statistical difference between groups *p *< 0.05 (Wilcoxon Test)

**Table 2:** Comparison of frequency of clinical characteristics in postmenopausal women (n = 225), according to bone mineral density (BMD)

Characteristics	N	BMD > -2 DP (n = 140)	BMD ≤ -2 DP (n = 85)	*p*-value*
Fall				0.055
Yes	130	64 (45.7)	66 (77.6)	
No	95	76 (54.2)	19 (22.3)	
Fall with fractures				0.054
Yes	21	8 (5.7)	13 (15.2)	
No	204	132 (94.2)	72 (84.7)	
Currently smoking				0.749
Yes	40	24 (14.7)	16 (18.8)	
No	185	116 (82.8)	69 (81.1)	
Use of hormone therapy				**< 0.001**
Yes	102	79 (56.4)	23 (27.0)	
No	123	61 (27.1)	62 (72.9)	
Physical exercise (PE)				0.618
Yes	76	49 (35.0)	27 (31.7)	
No	149	91 (65.0)	58 (68.2)	
Frequency of PE				0.893
Yes	46	32 (22.8)	14 (16.4)	
No	30	20 (14.2)	10 (38.8)	
Corrected Visual Deficit				0.495
Yes	194	119 (85.0)	75 (88.2)	
No	31	21 (15.0)	10 (11.7)	

*SD *standard-deviance

Data are expressed in numbers and in percentage between parentheses

*Statistical difference between groups *p *< 0.05 (Chi-Square Test)

**Table 3:** Comparison of stabilometric parameters in postmenopausal women (n = 225), according to bone mineral density (BMD)

Stabilometric parameters	BMD > -2.0 SD (n = 140)	BMD ≤ -2.0 SD (n = 85)	*p*-value*
Amplitude of X-axis oscillation latero-lateral (cm)	1.39 (1.02/1.97)	1.49 (1.09/2.08)	0.627
Amplitude of Y-axis oscillation antero-posterior (cm)	1.80 (1.42/2.61)	1.90 (1.52/2.52)	0.706
Oscillation area (cm^2^)	1.89 (1.21/4.04)	2.02 (1.41/3.61)	0.443

Values expressed as median with the 25th and 75th percentiles in parentheses

*Significantly different between groups (*p *< 0.05) (Wilcoxon Test)

It was observed that 57.8% (130/225) of the participants reported fall episodes in the past year without significant difference in the percent distribution between the groups (*p *= 0.055). Of these, 21 falls were followed by forearm (28.6%), ankle (23.8%), wrist (19.1%), foot (19.1%) and leg (9.4%) fracture. As regards the sites where the falls occurred, it was found that 63.1% (82/130) took place at home, 31.5% (41/130) in the street, and 5.4% (7/130) at work (Table 2). As to the occurrence of certain clinical characteristics, it was observed that, in the studied population, 17.8% were smokers (40/225), 86.2% showed visual deficit (194/225) with correction (wearing eyeglasses), 44.4% reported regular co-morbidities (100/225), 33.8% reported regular physical activity (76/225), of whom 39.5% (30/76) performed it ≥ 5 days/week, which did not differ between the groups (Table 2). It was found that 45.3% (102/225) were hormone therapy (HT) users, and among those with BMD > -2.0 SD, 56.4% used it whereas among those with BMD ≤ -2 SD, only 27.1% did, with significant difference between the groups (*p *< 0.05).

As regards stabilometric parameters, no differences were shown by the comparison between the groups. Higher dislocation amplitude (antero-posterior and latero-lateral) and in the dislocation area was observed for the group with BMD ≤ -2 SD when compared to that with BMD > -2.0 SD, although without statistical significance (Table 3). By evaluating the risk for falls in the presence of influential variables, it was observed that risk increased with age (OR 1.07; CI 95% 1.01-1.13), current smoking status (OR 2.19; CI 95% 1.22-3.21) and corrected visual deficit (OR 9.06; CI 95% 1.14-4.09). In contrast, HT use was associated with significantly reduced risk for falls (OR 0.48; CI 95% 0.26-0.88) (Table 4). Other clinical and anthropometric variables did not significantly influence risk for falls.

**Table 4:** Assessment of risk for falls in the presence of influential variables (postural parameters and clinical variables) in the 225 postmenopausal women

Variables	Odds ratio (OR)	CI 95%	*p*-value*
Age (years)	1.07	1.01-1.13	**0.013**
Current smoking	2.19	1.22-3.21	**0.042**
Use of Hormonal Therapy	0.48	0.26-0.88	**0.031**
Corrected Visual Deficit	9.06	2.55-32.18	**< 0.001**

*CI *confidence inteval. Only statistically significant results are presented. Other variables did show significance

*p < 0.05 (logistic regression)

## Discussion

The postural alterations that frequently occur with advancing age have led health care professionals to seek new forms for evaluating and identifying factors that contribute to functional deterioration. Evaluating postural balance and relating it to falls is essential to develop preventive and effective actions as well as to improve quality of life for postmenopausal women. Although there are few studies, the relationship between osteoporosis, fragility, falls and fractures has been increasingly acknowledged [3]. Although reveal a trend, the present study shows that the BMD was not associated with postural balance or fall risk parameters. Our study showed that 57.8% of women reported falls last 2 years and 16.2% of falls were followed fractures. Data in the literature indicate a frequency of falls from 30% to 60% for the evaluated patients [3,4,13] and that women are more likely to have a fall when compared men [20]. In fact, falls are responsible for 90% of the steady increase in hip fractures and the sixth cause of death among patients older than 65 years. Reducing risk for falls is a way to minimize the costs from care provision to the elderly, and it becomes possible as fall-determinant factors are identified [4].

Osteoporosis is responsible for body configuration changes that can interfere with postural control [12,13]. Our study found no direct relation between bone mass and fall occurrence. Abreu et al. [12] showed that women with normal BMD suffered more falls during the last 6 months than those with low BMD. According to those authors, this can be explained by the fact that their greater susceptibility to bone fractures among osteoporotic women makes them perform their daily activities tasks more carefully as well as use compensatory strategies to improve their postural balance [12]. Similarly, Smulders et al. [21], evaluating 85 elderly with osteoporosis, found that obstacle avoidance abilities were not impaired in persons with osteoporosis and they did not experience less balance confidence, measured with the short ABC-questionnaire, than the comparison older adults without osteoporosis [21]. On the other hand, Silva et al. [22] reported that the risk for falls in 133 women with osteoporosis, aged 60 years or greater, was twofold higher as compared to that in 133 women with normal bone mass [22]. It is known that osteoporosis can lead to spinal deformities such as thoracic kyphosis due to vertebral compression that alters the structure of the spine, causing weakness of the extensor muscles of the trunk and leading to reduced physical mobility and flexibility. Research shows that in severe kyphotic posture, the body moves forward leading to a shifting the center of balance, coming close to stability limits [3,13]. Given these effects on postural balance, patients who presented accentuated spinal deformities such as acute scoliosis and hyperkyphosis, were not included in our sample.

Sinaki et al. [1] have shown that osteoporotic elderly individuals have more corporal oscillation compared to those without osteoporosis. However, further explanation on the influence of osteoporosis on the balance and functional ability of elderly are lacking, particularly regarding gait, sitting down, and standing up. Although our study showed greater alteration for the stabilometric parameters in the group with low BMD, did not show significant differences between the groups. Previous studies showed similar results to ours [11,23]. Piirtola & Era [14] performed a systematic review of the use of the force platform as a fall indicator in older patients. The results suggest that some data from the force platform can be predictive of subsequent falls, especially as indicators of postural balance lateral control. However, due to the small number of studies included (only nine), it is difficult to reach a definite conclusion [14].

The risk factors for falls observed in this study were advancing age, visual deficit and current smoking. Age is an important factor that contributes to postural control alterations. In most studies on falls in older individuals, good performance was maintained until the age of 45-55 years, depending on the effort demanded. After such age range, a gradual deterioration in performance was observed [24]. Increased postural oscillation was shown in women older than 55 years, which is consistent with the increased number of forearm fractures [25]. Vision is most often needed for postural stability maintenance, particularly among older persons [2]. Lopez et al. [26] determined if there are associations between visual impairments and falls in 3014 women from the Australian Longitudinal Study on Women's Health. Postmenopausal women with reduced visual acuity were 1.82-fold more prone to suffer fall episodes and 1.79-fold more injuries from falls [26]. As regards smoking, it is believed that nicotine is related to alterations in vestibular functions. Previous study also reported balance alteration in smokers [27].

Studies suggest that hypoestrogenism would interfere with postural imbalance, explaining the increased occurrence of falls and fractures in the forearm in the menopausal period [28,29]. In our study, the hormone therapy use proved to be a protective factor in the risk of falls. These results are in agreement with those in the literature [25,29]. Naessen et al. [25] studied 40 healthy postmenopausal women randomized to receive 50 μg transdermal estradiol therapy or placebo for 6 months. During the balance evaluation, the force platform was used with measurements of postural oscillation speed. In HT users, balance improvement was observed, as shown by a 4.3% reduction in postural oscillation, which was a more significant response in women with lower basal estradiol values. In the placebo group, a 6.2% increase was found in oscillation. These authors discuss that the beneficial alterations in postural balance associated with HT are probably mediated through the brain and that such effect would be more evident if therapy were initiated in early postmenopause [25]. Bergström et al. [29] examined the effect of cyclic HT (estradiol valerate associated with medroxyprogesterone) and of physical exercise on postural balance by using the force platform for 60 perimenopausal women aged 44 to 51 years. After 18 months, HT users achieved better results for the postural balance parameters evaluated [29]. Despite the study's results indicate a probable protective effect of HT on risk of falls, the fact that women on HT were younger and with better BMD may have had some influence on such effect.

In agreement with the literature, in the present study, most falls (62.5%) occurred at home. Previous study showed that the site of highest fall incidence was, in fact, the household environment, from 53% to 60% of the episodes [30]. The fall is a multifactor event in which intrinsic (physiological) and extrinsic (environmental) factors have an influence. Among these, aspects such as shoe type, slippery surfaces, the presence of obstacles, objects on the floor and reduced illumination even in a familiar and well-known environment contribute to one's falling [31]. Hence, reducing fall risk is possible when the determinant factors of falls are identified and proper orientation is provided while addressing postmenopausal women.

This study shows some limitations in its interpretation. Because it is a cross-sectional investigation, the history of falls was obtained by means of semi-structured interviews that attempted to identify the number of falls retrospectively as well as complementary information such as the places of occurrence, environmental characteristics and the reason for falls, thus causing a recall bias that may have influenced the estimated frequency of falls. Another limitation is the small sample size. This can be attributed to the strict patient selection criteria, the period of application of balance tests that required trained professionals and to patients' refusal to perform the tests.

## Conclusions

Falls are multifactor events, and studies have been conducted in order to understand the relation of such variable to various factors; however, a definition has not yet been proposed in relation to the specific influence of extrinsic and intrinsic factors on falls or to which variables are the most important in such relation. In this scenario, studies must be performed in order to evaluate the possible factors related to falls so that strategies can be presented with the purpose to minimize their consequences, particularly the fractures in women with low BMD. From the present study, it can be concluded that in postmenopausal women, the BMD was not associated with postural balance or fall risk parameters. Age, smoking and corrected visual deficit were indicators of fall risk whereas HT use showed to be a protective factor.

## Competing interests

The authors declare that they have no competing interests.

## Authors' contributions

LC is the principal investigator and the principal author of the manuscript. JN-N and EPN are the supervisors of the principle investigator and responsible for the medical and scientific content of the manuscript. ABB, DB and GU have contributed substantial to the realization of the study. All the authors have read and approved the manuscript and agree with publication of their names.

## Funding

This study was supported by Sao Paulo Research Foundation (FAPESP), process number 2008/10378-2.

## Pre-publication history

The pre-publication history for this paper can be accessed here:

http://www.biomedcentral.com/1471-2474/13/2/prepub
